# Successful treatment of a severe Takotsubo syndrome case complicated by liver abscess

**DOI:** 10.1186/s12872-023-03145-7

**Published:** 2023-04-10

**Authors:** Zhi-Yue Zhang, Jin-Jin Sun, Jun-Hua Wang, Peng Wang, Bai-Mei Liu, Jun-Hua Xing, Jun Liu, Da-Peng Zhang, Zhen-Zhen Kong, Hai-Tao Zhang, Xin-Ya Yu

**Affiliations:** 1 Department of Cardiology, Air Force Medical Center, 100142 Beijing, China; 2 Hebei North University, Zhangjiakou, 075000 Hebei China; 3 Department of Research, Air Force Medical Center, 100142 Beijing, China

**Keywords:** Takotsubo syndrome (TTS), Left ventriculography, Intra-aortic balloon pump (IABP), Heart failure, Pump failure, Stress reaction, Liver abscess, Coronary arteriography (CAG)

## Abstract

The main manifestations of Takotsubo syndrome (TTS) are a spherical expansion of the left ventricle or near the apex and decreased systolic function. TTS is mostly thought to be induced by emotional stress, and the induction of TTS by severe infection is not often reported. A 72-year-old female patient with liver abscess reported herein was admitted due to repeated fever with a history of hypertension and impaired glucose tolerance. Her severe infection caused TTS, and her blood pressure dropped to 80/40 mmHg. IABP treatment was performed immediately and continued for 10 days, and comprehensive medication was administered. Based on her disease course and her smooth recovery, general insights and learnings may be: Adding to mental and other pathological stress reaction, serious infections from pathogenic microorganism could be of great important causation of stress reaction leading to TTS, while basic diseases such as coronary heart disease, hypertension, and diabetes were be of promoting factors; In addition to effective drug therapies for TTS, the importance of the timely using of IABP should be emphasized.

## Introduction

Takotsubo syndrome (TTS) is also known as apical ballooning syndrome and octopus trap syndrome. TTS was first proposed by Japanese scholar Sato and his colleagues in 1990 [1]. TTS mainly manifests as spherical dilation and wall motion abnormalities in the apex of the heart and in the middle of the left ventricle near the apex of the heart and decreased myocardial contractility. Most patients have symptoms similar to those of acute coronary syndrome(ACS), but do not have coronary artery syndrome blockage, ST-segment elevation can be observed on an electrocardiogram. The overall incidence of TTS is higher among women than men, especially among postmenopausal women. The possible complications of TTS often include acute pulmonary oedema, significant left ventricular insufficiency, mitral valve regurgitation caused by forwards movement of the mitral valve and chordae tendineae, left ventricular mural thrombosis, left ventricular free wall rupture, acute pericarditis, ventricular septal perforation, ventricular tachycardia (torsade de pointes), ventricular fibrillation, and even cardiogenic shock and leading to death [2–7]. Moreover, the induction of TTS is mostly considered to be related to emotional stress, and TTS induced by severe infection is not often reported [8, 9]. The relationship between the occurrence of the disease and the underlying disease is also not discussed much. The treatment of TTS commonly emphasizes comprehensive drug treatment and indications and the period of treatment for mechanotherapy, such as intra- aortic balloon pump (IABP), have not been extensively discussed.

## Case data

A 72-year-old female patient was admitted to the hospital due to intermittent fever for 6 days. She had a history of hypertension for 10 years, her highest blood pressure was 180/90 mmHg, and she had “impaired glucose tolerance” more than six months. She had retired for more than 10 years, her family was in a good financial shape, had good relationship with family members and friends, and had no mental or emotional anamnesis. The patient had recurrent fever without an obvious cause. Her body temperature fluctuated between 38–40 °C, and her highest body temperature was 40 °C. Before admission, she received oral and intravenous antibiotics and antipyretic treatment with ibuprofen and was admitted to the hospital due to lack of clinical relief.

**Fig. 1:**
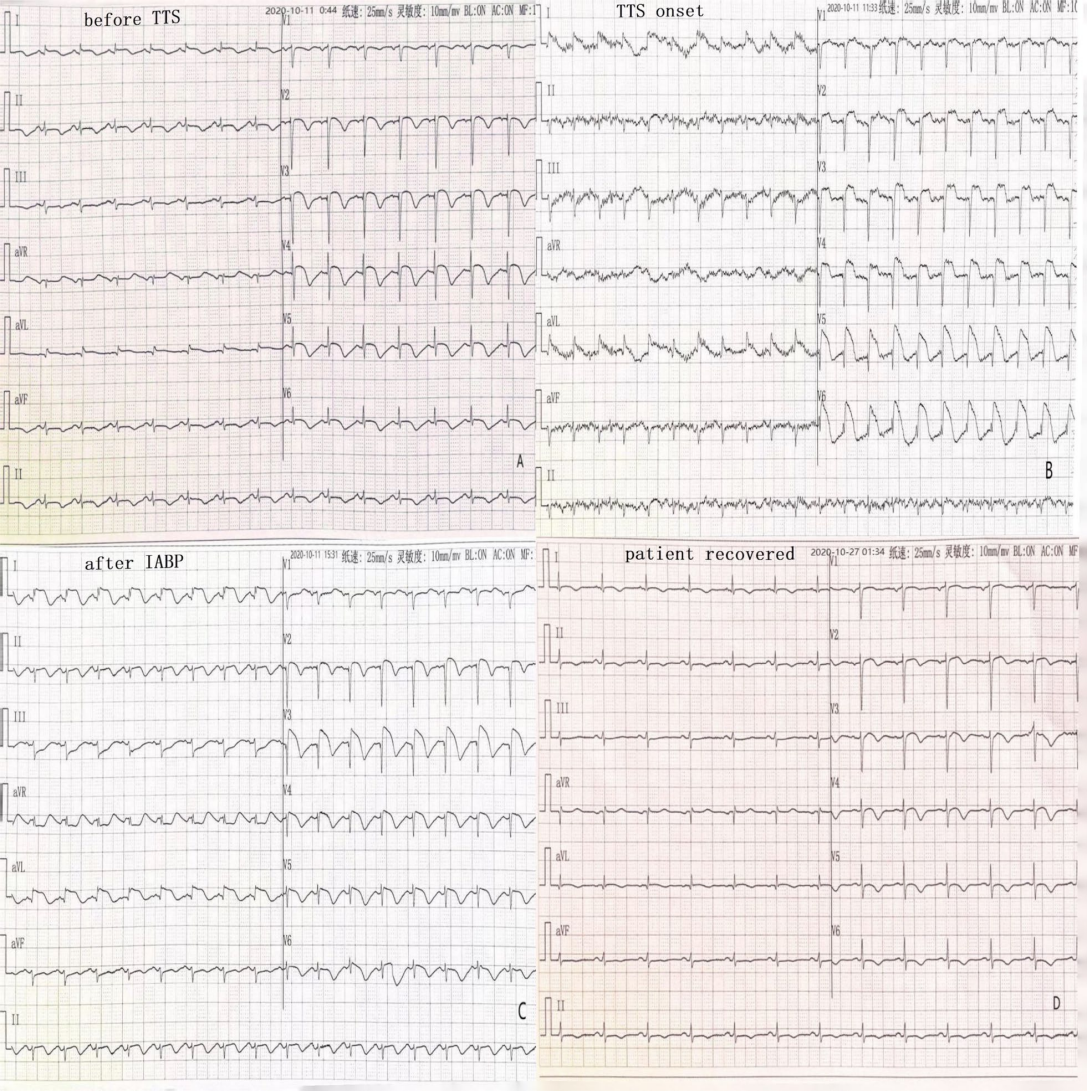
Eelectrocardiograms during the disease course. The first one recorded at admission, when TTS onset, extensive anterior wall arched ST-segment elevations, and it recovered a lot soon after IABP, and completely restored 2 weeks later

On the day of admission, her physical examination showed that her body temperature was 36℃, pulse was 86 beats/min, and blood pressure was 105/56 mmHg, with face of acute ill, intermittent lethargy, and a small amount of wet and dry rales and wheezing in both of her lungs. Her heart rate was 86 beats/min, no murmur was heard around the area of any of her valves, and the rhythm was uniform. Mild pitting oedema was noted in her lower limbs and hands. Her laboratory examination showed increased white blood cells, percentage neutrophils, rapid C-reactive protein, procalcitonin, and blood sugar, but there were decreases in her red blood cell count, haemoglobin, platelet count, sodium ion and plasma protein. Her B-type natriuretic peptide (BNP) was 2717.7 pg/ml on admission, which was lower than before days (13,404 pg/ml) but was still 27 times the normal value. Increased levels of myocardial injury markers troponin I (cTnI), creatine kinase isoenzyme (CK-MB) and myoglobin were noted. An electrocardiogram showed a sinus rhythm with I, II, aVF, and V2-V6 lead T wave inversions (Fig. 1 before TTS). Ultrasound cardiogram showed that the movement amplitude of part of the ventricular septum, anterior wall and sidewall was reduced, the movement of the ventricular wall was not coordinated, and her heart function was reduced. There was no typical spherical dilation of the left ventricle. Abdominal computed tomography (CT) showed a fatty liver and an abnormal density in the right lobe of the liver. A chest X-ray showed signs of cardiac insufficiency. The electrocardiogram results were consistent with the myocardial enzyme spectrum examination, and the possibility of myocardial infarction was considered. The patient was administered routine dual antiplatelet therapy (DAPT) with aspirin and clopidogrel. In addition, the patient was given creatine phosphate nutrition myocardial therapy and fondaparinux sodium via subcutaneous injection. She was given furosemide injection as a diuretic therapy. Then, based on her assay results, tolvaptan was administered, and sodium chloride and albumin were supplemented. The patient had an intermittently high fever, and laboratory examinations found subsequent increases in the routine white blood cell count, percentage of neutrophils, rapid C-reactive protein and procalcitonin. These results showed the presence of a bacterial infection, and abdominal CT suggested liver internal space-occupying changes, especially considering that the earlier identified liver abscess may be large, and no other signs of infection, such as in the lungs and urinary tract were found. Combined anti-infective therapy of cefoperazone sodium and sulbactam sodium injection with levofloxacin sodium chloride injection were given. Before admission she had elevated fasting blood glucose, but had no drug treatment. During hospital, her blood glucose was as high as 30.1 mmol/L, therefore, treated with insulin aspart and insulin glargine injection.

On the second day, with no emotional reason or other disease causing, her sudden sweating and wheezing appeared, with heart rate increased to 150 beats/min, and blood pressure increased to 203/110 mmHg; but the blood pressure dropped to 80/40 mmHg soon. An urgent ECG examination revealed extensive anterior wall arched ST-segment elevations (Fig. 1 TTS onset). She had haemodynamic disorders, the blood pressure was too low and difficult to maintain normal by medications. However, according to clinical data of the time, it was difficult to determine whether she suffered acute extensive anterior wall myocardial infarction or TTS; nevertheless, the use of intra-aortic balloon counter pulsation (intra-aortic balloon pump, IABP) won’t be conflicting, so IABP was implemented immediately. After successful installation, with its starting work, the blood pressure rose to 95/65 mmHg, and her symptoms relieved soon after. Specifically, rechecked BNP significantly lowered to 1641.6 pg/ml. Afterwards, medications such as anti-infection, anti-platelet, diuretic, protein supplement, continued, and her condition became stable.

**Fig. 2:**
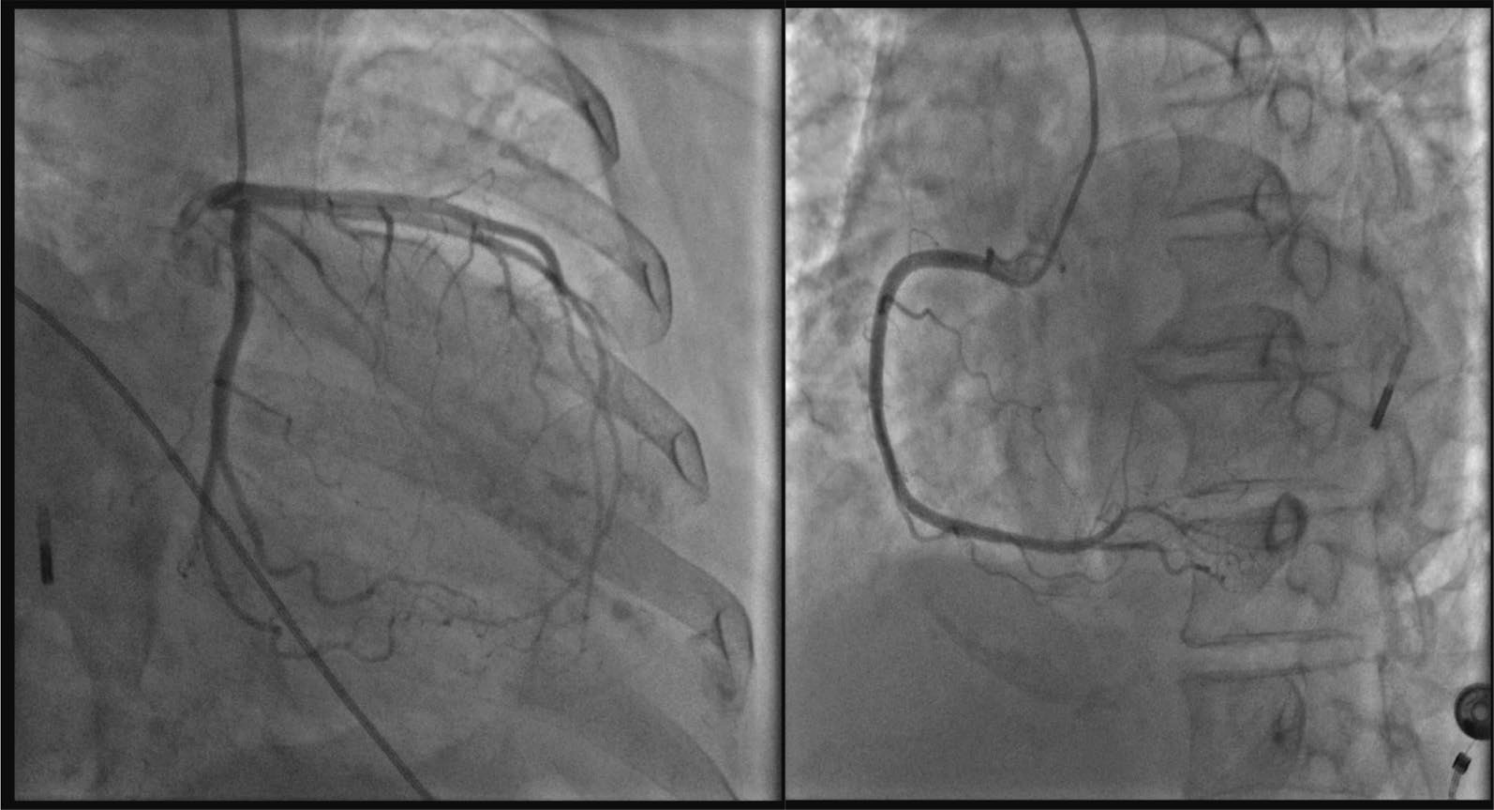
Coronary angiography images (LCA left, RCA right). All the coronary artery branchs had no obvious stenosis

**Fig. 3:**
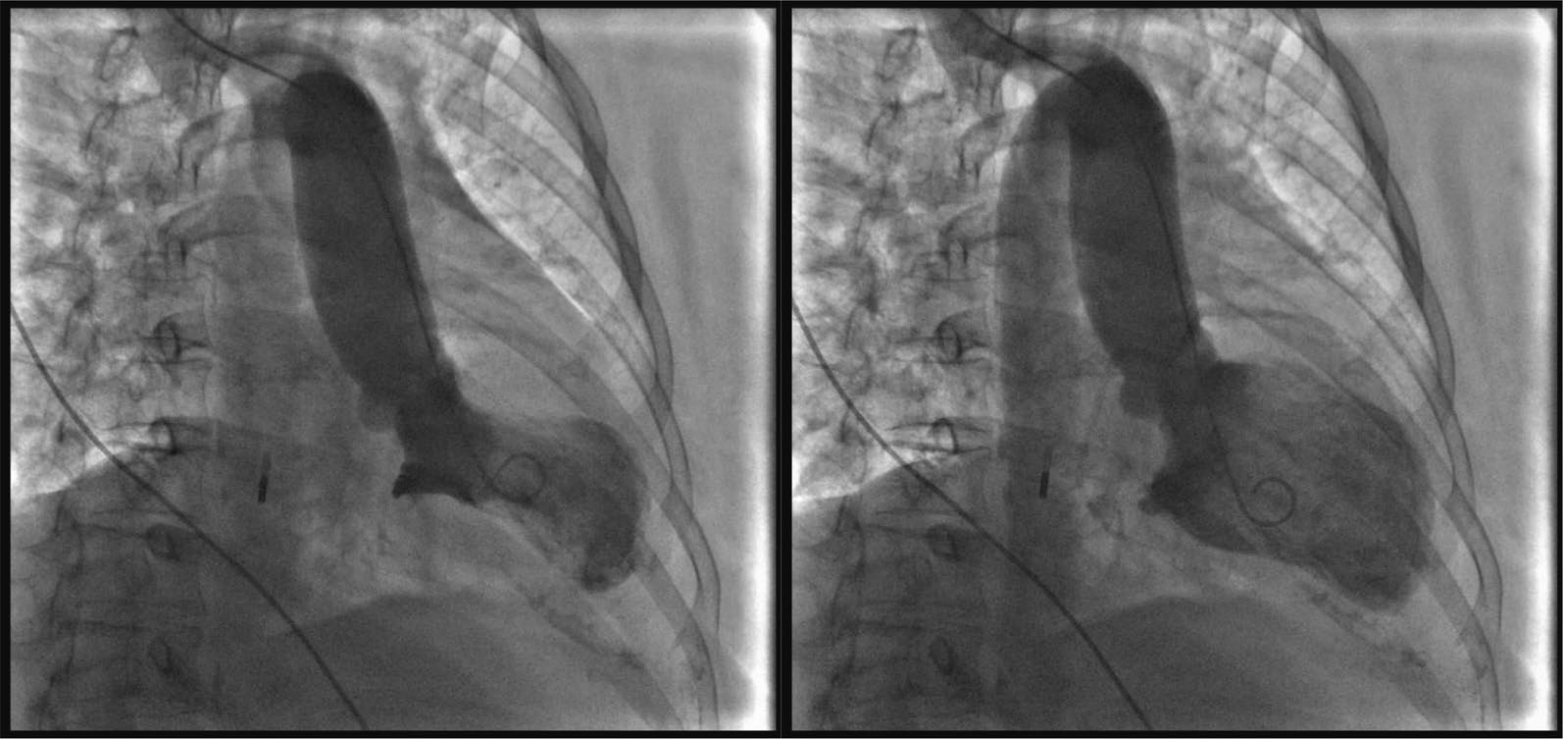
Left ventriculography images (systole left, diastole right). The anterior wall of the left ventricle had no movement, the apex of the heart showed a typical spherical dilation, the contraction movement of the bottom of the heart was normal, and the left ventricular ejection fraction was 47%

On the eleventh day, when her CK and CK-MB returned to normal, coronary angiography and left ventriculography was performed. The results showed that all the coronary artery branchs had no obvious stenosis (Fig. 2), the anterior wall of the left ventricle had no movement, the apex of the heart showed a typical spherical dilation, the contraction movement of the bottom of the heart was normal (Fig. 3), and the left ventricular ejection fraction was 47%. The angiographic results excluded acute myocardial infarction caused by coronary artery obstruction, and the diagnosis of Takotsubo syndrome (TTS) was confirmed.

During hospital, she had a recheck abdominal B-ultrasound. The results showed that there was a round low-density shadow in the upper posterior part of the right liver lobe, and this lesion was diagnosed as a liver abscess. After active anti-infection treatment, the abscessed area did not spread, liquefy or form a cavity and gradually absorbed, and finally cured. After successful combined treatment of medication with IABP, the ECG ST-segment elevation gradually returned to normal (Fig. 1 after IABP), and her T waves gradually returned to normal, only a shallow inversion left (Fig. 1 patient recovered). As the clinical symptoms disappeared, she discharged from the hospital.

## Discussion

In this case, after active and effective treatment, her severe cardiac insufficiency was controlled in time, and she was discharged from the hospital smoothly. She did not develop any other severe complications. However, the clinical manifestations and various examinations of TTS in this case met the early Mayo Clinic diagnostic criteria and the latest international expert consensus on TTS [10–12]. This case may have implications for clinical work in the following ways.

Regarding the pathophysiology of TTS, previous studies have shown that coronary microvascular dysfunction and coronary artery spasm are important pathophysiological mechanisms in this disease [13]. The prominent clinical feature of this case was that the severe disease course started with severe infectious disease, which manifested as a liver abscess, and the patient had an intermittently high fever and increased CRP. B-Ultrasound showed that the area of the liver abscess was extremely large. These results revealed that severe infection could cause serious cardiac complications, such as TTS. The patient had several comorbidities, including hypertension and diabetes. These chronic diseases can cause damage to vascular endothelial cellular function, and sepsis itself can not only aggravate vascular endothelial damage but also directly damage myocardial cells [14]. Moreover, severe infections lead to systemic inflammation, which directly damages the cellular function in various organs. At the same time, infection causes a systemic stress response and aggravates damage to the vascular endothelium. Moreover, it also activates the sympathetic nervous system, increasing the level of catecholamines in the blood, and causing blood vessel contraction [15–17]. This causes an increase in myocardial contractility, which increases myocardial oxygen consumption and possibly even causes extensive coronary spasm and therefore myocardial ischaemic damage. Catecholamines could also lead to an increase in peripheral vascular tone and even sudden elevation in blood pressure, leading to a sudden increase in the afterload of the left ventricle and aggravating myocardial damage. Ultimately, decompensated expansion of the left ventricle and pump failure can occur.  And this claim was verified in numerous COVID-19 cases [9]. In addition, this case was a postmenopausal woman prone to TTS, as vascular endothelial cells did not have access to the protective effect of oestrogen. Furthermore, this patient had obvious hyponatremia due to low food intake, fever and consumption, which affected the cellular function of various organs, including myocardial cells. This was also an important cause of the TTS in this case. Acute myocardial infarction, severe heart failure caused by TTS, or other damaging factors mentioned above could all lead to an increase in myocardial enzymes, so these findings had no distinguishing diagnostic value here. In this case, the lowered ejection fraction shown by echocardiography and the increased BNP seen in the laboratory tests showed that there was obvious heart failure. After analysing this case, it could not be ruled out that the acute extensive anterior myocardial infarction that actually occurred was caused by coronary spasm, and on this basis, TTS syndrome occurred due to the above factors.

The diagnosis of TTS is dependent on echocardiography or left ventricular angiography to see the “octopus tank” spherical dilation of the left ventricle while excluding obvious coronary stenosis. In this case, the patient had risk factors for coronary artery disease, such as hypertension and diabetes. After the onset of ST segment depression and T wave inversion, as noted on the ECG, when the patient’s disease worsened, extensive arched ST-segment elevations occurred on anterior wall leads, accompanied by a significant increase in myocardial enzymes. In the early period, reduced ventricular wall motion and reduced heart function were observed on echocardiography, but no typical “octopus tank”-like spherical expansion of the left ventricle was observed. Therefore, although TTS could not be ruled out, the clinical diagnosis of acute myocardial infarction from coronary heart disease could also be logical. Because the emergency coronary intervention window period had passed, IABP and continuing drug therapy were given to the patient according to conventional principles. Coronary angiography and left ventricular angiography were performed after the patient’s condition was stable. Typical spherical dilation of the left ventricle was seen, with no obstructive coronary lesions, so the diagnosis of TTS was established. As coronary angiography and left ventricular angiography are not commonly performed in similar cases in clinical practice, the diagnosis process of this case suggested that the number of missed diagnoses of TTS might be significant.

Regarding the therapy strategies, in addition to conventional anti-infection therapy and the control of inducing factors, blood sugar and blood pressure, the important treatment measures for this patient involved haemodynamic correction, antiplatelet therapy and anticoagulation. IABP increases the coronary perfusion pressure by increasing the diastolic pressure in the aorta, which reduces coronary vascular dysfunction and decreases the left ventricular systolic internal aortic pressure to increase the cardiac output, thereby reducing the left ventricular end-diastolic pressure and myocardial oxygen consumption [18]. This emergency measure that was given to improve the haemodynamic function of the ventricle improved the pump failure caused by acute myocardial infarction or TTS. Therefore, as in this case, when it was difficult to distinguish between acute myocardial infarction or TTS, a timely application of IABP was not contradictory, and the using duration of IABP should be based on the patient’s haemodynamic recovery. In this patient, the using duration of IABP was 10 days, and the patient ultimately recovered well. In addition, for acute myocardial infarction or acute coronary syndrome, according to the guidelines, antiplatelet and heparin anticoagulation therapy should be routinely utilized. For TTS patients, as complicated ventricular mural thrombosis may be possible, the use of antiplatelet and anticoagulation agents also has preventive effects. Therefore, the treatment strategies for the two diseases are concordant, so, if there is no contraindication, antiplatelet and anticoagulant treatments should be used in time even if the diagnosis of acute myocardial infarction or TTS cannot be confirmed.

## Conclusion

The patient underwent active IABP treatment and comprehensive drug therapy, did not develop any severe complications, and was discharged with a good recovery. After analysing the relevant literature and the treatment experience of this case, it enlightened us regarding TTS in four ways. ① Regarding the pathophysiology, severe infections such as liver abscesses can induce TTS, and underlying diseases, such as coronary heart disease, hypertension and diabetes, are of promoting factors for the pathogenesis of TTS because these conditions may predispose patients to coronary spasm. ② As coronary angiography and left ventricular angiography are not commonly performed in similar cases in clinical practice, it could be inferred that the rate of missed diagnosis of TTS cases might not be too low; therefore, the actual morbidity of TTS may be much higher than what is clinically reported. ③ Regarding the treatment of TTS, especially when the diagnosis is not definitive between acute myocardial infarction and TTS, if there are no contraindications to treatment, antiplatelet and anticoagulation therapy should be used in time and it  is very important to give IABP treatment in time.

## Data Availability

The datasets used and analyzed during the current study are available from the corresponding author on reasonable request.

## Abbreviations

BNP: Brain natriuretic peptide
ECG: Electrocardiogram
TTS: Takotsubo syndrome
IABP: Intra-aortic balloon pump
CAG: Coronary arteriography
DAPT: Dual antiplatelet therapy
CT: Computed tomography
cTnI: Troponin I
CK-MB: Creatine kinase isoenzyme
